# Cumulative atherosclerosis index of plasma exposure and new-onset diabetes in middle-aged and older adults: a prospective cohort analysis from the China Health and Retirement Longitudinal Study

**DOI:** 10.3389/fnut.2025.1653764

**Published:** 2025-10-29

**Authors:** Yu Li, Rong Gu, Li Chen, Qi Zhao, Yue Wang

**Affiliations:** Nanjing University of Chinese Medicine, Nanjing, Jiangsu, China

**Keywords:** cumulative atherosclerosis index of plasma exposure, new-onset diabetes, dose-response relationship, restricted cubic spline regression, prospective cohort study

## Abstract

**Introduction:**

China bears the world's largest diabetes burden (116 million adults). The Cumulative Atherosclerosis Index of Plasma (CumAIP), reflecting lipid-driven atherogenicity, may contribute to diabetes pathogenesis. This study investigates longitudinal associations between cumulative CumAIP exposure and diabetes incidence in middle-aged and older Chinese adults.

**Method:**

Using data from the China Health and Retirement Longitudinal Study (CHARLS), we analyzed 10,395 diabetes-free participants at baseline (2011) with follow-ups in 2013, 2015, and 2018. Multivariable logistic regression adjusted for sociodemographic (age, gender, education), lifestyle (smoking, alcohol, sleep, physical activity), and clinical factors (systolic and diastolic blood pressure, BMI, waist circumference). Restricted cubic splines assessed dose-response relationships.

**Result:**

Over 7 years, 793 participants (15.73%) developed diabetes. The highest CumAIP quartile (Q4) had a 3.43-fold elevated diabetes risk vs. Q1 (OR = 3.82, 95% CI: 3.13–4.67). A linear dose-response relationship was observed (p for nonlinearity=0.44); each interquartile increase in CumAIP above 1.03 elevated risk by 90% (OR = 1.90, 95% CI: 1.75–2.07). CumAIP predicted diabetes moderately (AUC=0.64, cutoff = 1.03).

**Conclusion:**

Cumulative CumAIP exposure independently predicts diabetes incidence in middle-to-older adults, highlighting its potential for clinical risk stratification.

## 1 Background

The Growing Burden of New-onset Diabetes in Aging Populations: An Emerging Public Health Challenge. The incidence of this disease has been going up on a global scale. It is reported that the global prevalence of diabetes in 2019 is estimated to be 9.3%, rising to 10.2% by 2030 and 10.9% by 2045 (1). China bears the heaviest burden, accounting for over 25% of global cases, where approximately 116 million adults live with diabetes (2). Therefore, identifying potential risk factors is of vital importance, which can provide strategies for prevention in medical practice.

The cumulative atherosclerosis index of plasma (CumAIP), calculated as log(triglycerides/HDL-C), quantifies long-term exposure to atherogenic lipid profiles. Unlike single-timepoint measurements, CumAIP integrates longitudinal lipid variations, potentially offering superior predictive value for diabetes onset (3). AIP is used to assess an individual's risk of cardiovascular diseases and other conditions, especially the risk of atherosclerosis. Its calculation is based on the ratio of triglycerides (TG) to high-density lipoprotein cholesterol (HDL-C) in plasma. CumAIP is a more comprehensive indicator aimed at evaluating the cumulative impact of lipids in plasma on diseases such as atherosclerosis.

Atherosclerosis may also be a factor contributing to diabetes, as it has been found that the atherosclerotic burden exists before the onset of diabetes, accompanied by pro-atherosclerotic inflammation and vasoconstriction (4, 5). In terms of lipid patterns, individuals with prediabetes usually exhibit atherosclerotic dyslipidemia, characterized by elevated triglycerides (TG) and decreased very low-density lipoprotein cholesterol (vLDL-C) and high-density lipoprotein cholesterol (HDL-C) (6–10). CumAIP is closely associated with the development of prediabetes (11–13).

However, the longitudinal impact of cumulative atherogenic burden (quantified as CumAIP) on diabetes incidence in aging populations remains unestablished. To address this knowledge gap, we analyzed CHARLS cohort data to evaluate clinically relevant associations between CumAIP trajectories and diabetes development.

## 2 Method

### 2.1 Study population

This study is an observational cohort study. The subjects were from the National Longitudinal Study of Health and Aging in China (CHARLS), which is a nationally representative longitudinal survey in China (14). CHARLS uses structured survey tools to collect comprehensive data by conducting face-to-face interviews with Chinese people aged 45 and above. This survey includes standardized measurements of sociodemographic factors, lifestyle variables and health-related information. In 2011, CHARLS recruited subjects from 10,257 families in 150 counties, districts and 450 towns of 28 provinces in China. A follow-up assessment is conducted every 2 years. The data was weighted to ensure that the sample accurately represents the national population. More detailed information about the CHARLS method has been reported previously. CHARLS is a reliable database for studying the health status and possible influencing factors of the middle-aged and elderly population.

In this prospective cohort study, we analyzed baseline data in waves 2011, 2013, 2015 and 2018 from CHARLS (http://charls.pku.edu.cn/), a freely accessible database of a nationally representative sample. The following inclusion criteria were implemented for this study based on the research purpose: age ≥ 45 years old; Complete sociodemographic data, including gender, educational level, marital status and location; And the complete data of fasting blood glucose. Participants with a history of taking lipid-lowering drugs were excluded. Ethical approval for the CHARLS cohort was granted by Peking University's Ethics Review Committee (IRB00001052-11015), with written informed consent obtained from all participants prior to enrollment.

**Figure 1:**
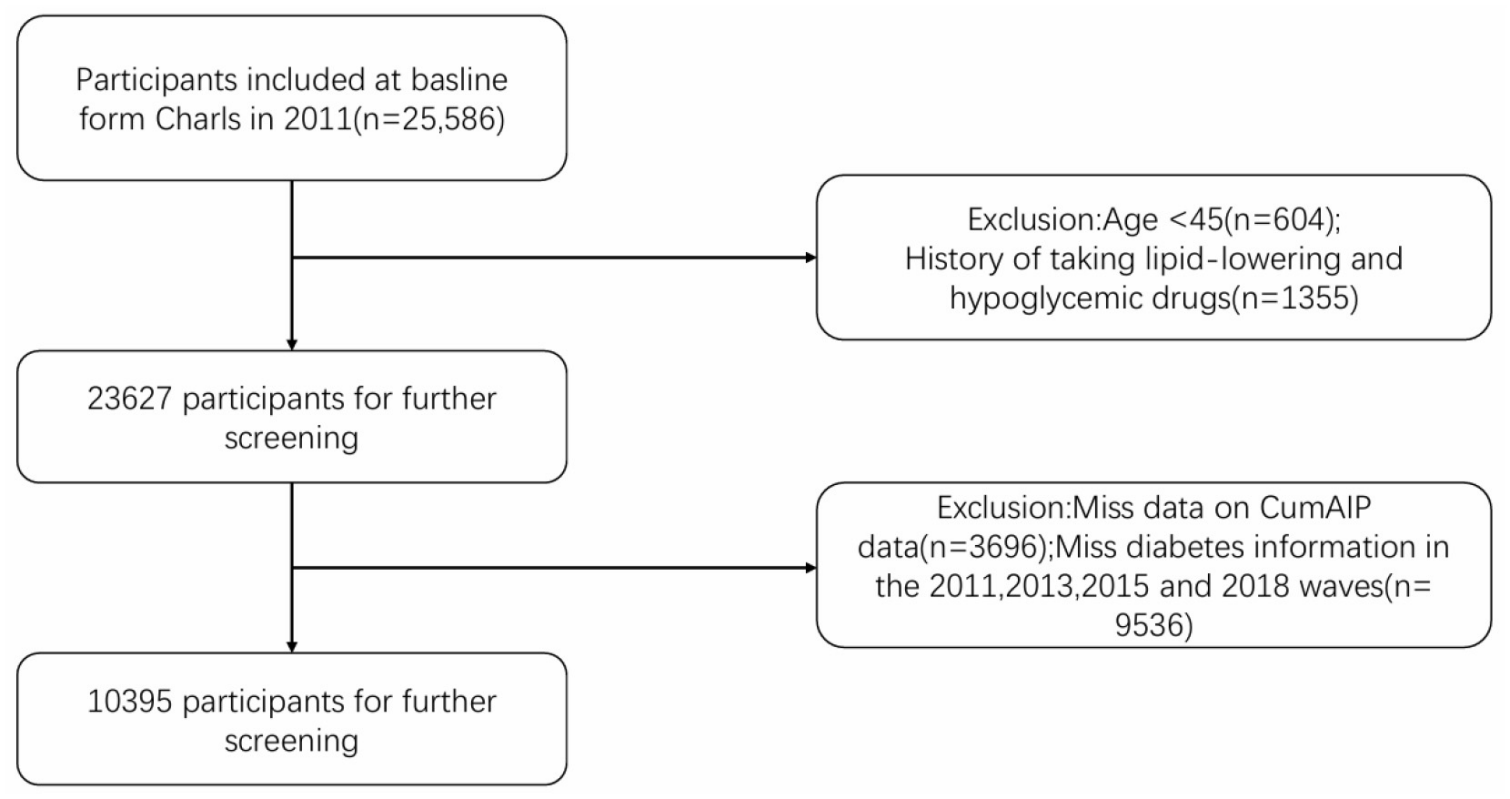
Flowchart detailing the process of participant selection.

Figure 1 shows a flowchart detailing the process of participant selection. Among the participants of the baseline survey in 2011, 10,395 people completed physical examinations and questionnaire evaluations. Among these participants, they were subsequently excluded from the study based on the following specific criteria: Age under 45 years old (604 individuals), history of lipid-lowering and hypoglycemic drug use (1,355 individuals), incomplete CumAIP data (3,696 individuals), lack of diabetes information in 2011, 2013, 2015, 2018 (5,280 individuals), and 843 individuals diagnosed with diabetes at baseline. Ultimately, a total of 8,754 eligible participants were included. Among them, there were 646 new-onset diabetes in 2013, 137 in 2015 and 10 in 2018. The history of lipid-lowering and hypoglycemic drug use was obtained based on the self-reports of the participants. In the CHARLS questionnaire, the relevant question raised is, “Are you currently undergoing any of the following treatments for [dyslipidemia or diabetes or hyperglycemia] or its complications (select all applicable items)?” Options include taking Western medicine, traditional Chinese medicine or other treatment methods. The history of lipid-lowering and hypoglycemic drug use was obtained based on the self-reports of the participants. In the CHARLS questionnaire, the relevant question raised is, “Are you currently undergoing any of the following treatments for [dyslipidemia or diabetes or hyperglycemia] or its complications (select all applicable items)?” Options include taking Western medicine, traditional Chinese medicine or other treatment methods.

### 2.2 Calculation of CumAIP

The cumulative atherogenic index of plasma (CumAIP) was calculated using the trapezoidal rule for area under the curve. For the two measurement timepoints (2012 and 2015), the formula was:


$$\begin{array}{l} \text{CumAIP}=\left[\left({\text{AIP}}_{\left(2012\right)}+{\text{AIP}}_{\left(2015\right)}\right)\times \;\left(2015-2012\right)\right]/2 \end{array}$$

where AIP_(2012)_ and AIP_(2015)_ represent the atherogenic index values at baseline and follow-up, respectively, and the time interval (2015–2012) equals 3 years. This formulation is mathematically equivalent to the rectangular area under the curve method in the two-timepoint context, as both approaches reduce to linear combinations of the two AIP measurements.

Sensitivity analyses revealed that four alternative formulations of cumulative AIP exposure (trapezoidal AUC, rectangular AUC, time-weighted average, and simple mean) were perfectly correlated (r = 1.000) with the primary metric, indicating mathematical equivalence in the context of two measurement timepoints. The follow-up-only approach demonstrated high but imperfect correlation (r = 0.875), supporting the value of incorporating baseline measurements.

### 2.3 Determination and definition of new-onset diabetes and its development

The diagnosis of new-onset diabetes was based on self-reported data. According to the standards published by American Diabetes Association in 2005, this study defined NDM as a fasting blood glucose level ≥126 mg/dl (7 mmol/L), and/or a random blood glucose level ≥200 mg/dl (11.1 mmol/L), and/or an HbA1c level ≥6.5%, and/or self-reported diagnosis with yes (“Have you ever been diagnosed with diabetes or hyperglycemia?”) Subjects with diabetes in 2011 were excluded. If a patient was diagnosed with diabetes during the follow-up period in 2013, 2015, and 2018, he or she was included in the study according to our definition of newly diagnosed diabetic patients. Venous blood was drawn after a 12-h overnight fast. On-site complete blood count testing was performed immediately. Whole blood samples were temporarily stored at 4 °C before being transported to a central laboratory for analysis. Glucose, total cholesterol (TC), triglycerides (TG), low-density lipoprotein cholesterol (LDL-C), and high-density lipoprotein cholesterol (HDL-C) levels were measured using enzymatic colorimetric assays. Glycated hemoglobin (HbA1c) was quantified by boronate-affinity high-performance liquid chromatography.

### 2.4 Assessment of covariates

The analyses were adjusted for sociodemographic characteristics, health-related behaviors, and anthropometric measurements. We analyzed age, gender, education level (“primary school or below”, “high school”, and “college or above”), location (“city/town” and “village”), and marital status (“married” and “never-married/separated/widowed”) as demographic variables. We analyzed smoking status (“non-smoker”, “ex-smoker”, and “current smoker”), drinking status (“never”, “less than once a month”, and “more than once a month”), sleep time and physical activity as variables of health-related behavior. Those data were obtained from self-reported questionnaires and with the help of trained interviewers. Laboratory test results included triglycerides, glucose, and body mass index (BMI), waistline (WC), high-density lipoprotein (HDL).

### 2.5 Statistical analysis

**Figure 2:**
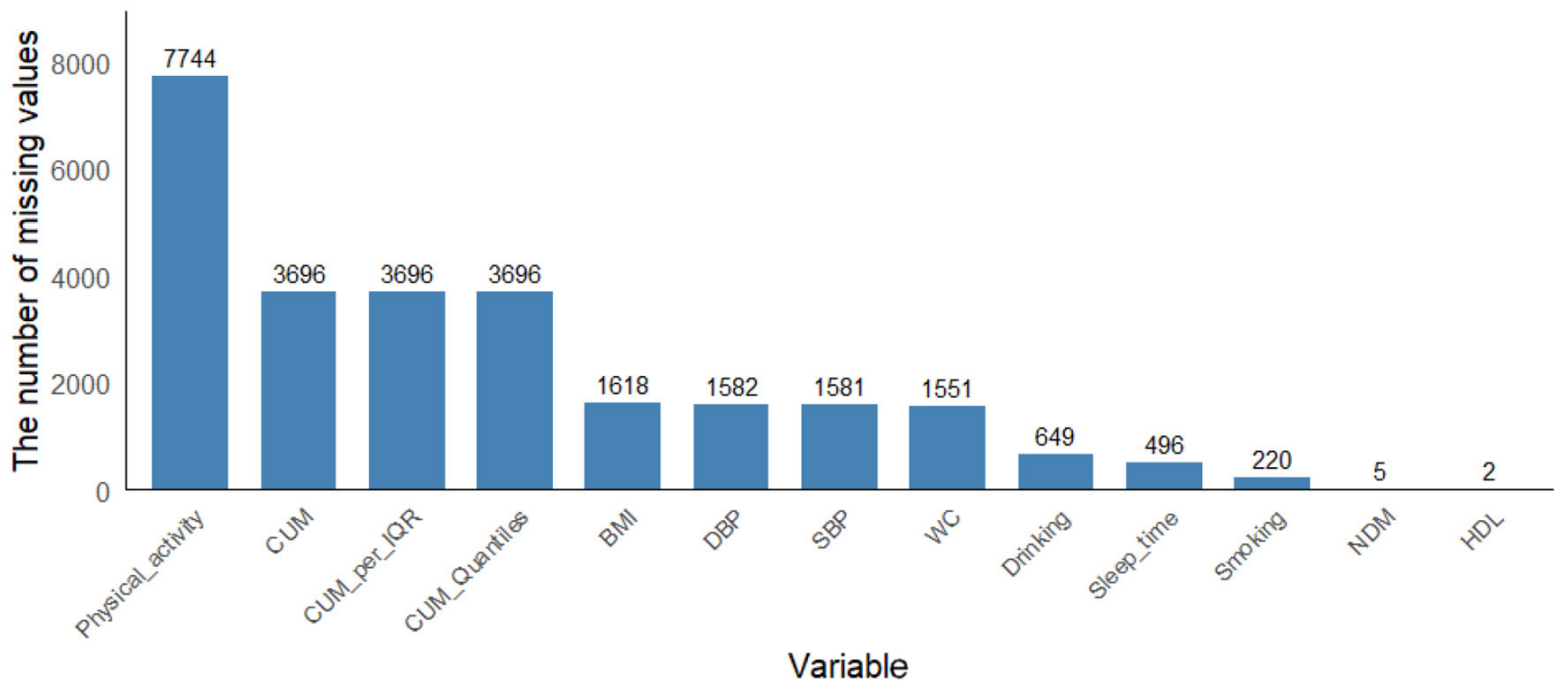
The result of re-interpolating the missing values after data screening.

To address potential selection bias arising from the exclusion of 17,561 participants (68.6% of the initial sample) due to missing follow-up blood glucose data, we performed multiple imputation using the Multivariate Imputation by Chained Equations (MICE) approach via the mice package (version 4.4.3) in R. Figure 2 is the result of re-interpolating the missing values after data screening. The imputation model included all variables used in the primary analysis: demographic characteristics (age, gender, education, location, marital status), health behaviors (smoking, drinking, sleep duration and physical activity), clinical measurements (systolic and diastolic blood pressure, BMI, waist circumference), and laboratory values (triglycerides, HDL, LDL, total cholesterol, HbA1c). We generated 5 imputed datasets using predictive mean matching for continuous variables and logistic regression for categorical variables, with 10 iterations per imputation to ensure convergence. The random seed was set to 1,234 to ensure reproducibility. We subsequently applied our primary analytic models to each imputed dataset and pooled the results using Rubin's rules to obtain final estimates that account for both within-imputation and between-imputation variability.

Continuous variables are presented as mean ± standard deviation for normally distributed data or median (interquartile range) for skewed distributions, while categorical variables are expressed as percentages. Baseline characteristics and diabetes incidence across CumAIP quartiles (Q1–Q4) were compared using one-way ANOVA, Kruskal-Wallis H test, or χ^2^ test as appropriate. Three hierarchical logistic regression models estimated the odds ratio (OR) and 95% confidence interval (CI) for diabetes risk: Model 1 (unadjusted), Model 2 (adjusted for sociodemographic factors: age, gender, education, location, marital status), and Model 3 (further adjusted for health behaviors: smoking, drinking, sleep duration), with CumAIP analyzed both continuously (per IQR increment) and categorically (quartiles). Predictive performance was assessed through receiver operating characteristic (ROC) curve analysis (AUC calculation), with the optimal cutoff determined using Youden's index. Dose-response relationships were evaluated using restricted cubic splines (RCS), and interaction effects were tested by incorporating product terms [CumAIP × modifier] for sociodemographic, behavioral, and anthropometric variables. All analyses were conducted in R 4.4.3 (rms package for RCS; rpart for decision trees), with statistical significance defined as two-tailed *P* < 0.05.

## 3 Result

### 3.1 Characteristics of the study participants according to the new-onset diabetes and CumAIP

The characteristics of the study population are shown in Table 1. A total of 10,395 participants were included (median age = 59.1, male = 4,949) (47.6%), female = 5,446 (52.4%), among whom 1,636 (10.13%) developed diabetes. The baseline median (IQR) CumAIP of all participants was 1.1 (0.8). The characteristics of newly diagnosed diabetes are significantly different from those of participants with diabetes. Specifically, the former is mostly female in gender, with higher SBP, DBP, BMI, WC, glucose, TG and CumAIP, and lower HDL-C.

**Table 1:** Summary of baseline characteristics of the study population according to CumAIP quartile group.

**Characteristic**	**Overall (*n* = 10,395)**	**Diabetes (*n* = 8,754)**	**NDM (*n* = 1,636)**	***P*-value**
**Demographics**				
Age, years, mean (SD)	59.1 (9.5)	59.0 (9.6)	59.5 (8.8)	0.082
Female, *n* (%)	5,446 (52.4)	4,493 (51.3)	952 (58.2)	< 0.001
**Education level**, ***n*** **(%)**				0.042
College or above	370 (3.6)	295 (3.4)	75 (4.6)	
High school	2,831 (27.2)	2,377 (27.2)	451 (27.6)	
Primary school or below	7,194 (69.2)	6,082 (69.5)	1,110 (67.8)	
**Location**, ***n*** **(%)**				< 0.001
City/town	1,929 (18.6)	1,534 (17.5)	393 (24.0)	
Village	8,466 (81.4)	7,220 (82.5)	1,243 (76.0)	
**Lifestyle factors**				
**Smoking status**, ***n*** **(%)**				< 0.001
Current smoker	3,060 (30.1)	2,681 (31.3)	377 (23.5)	
Ex-smoker	868 (8.5)	713 (8.3)	155 (9.7)	
Non-smoker	6,247 (61.4)	5,171 (60.4)	1,073 (66.9)	
**Drinking status**, ***n*** **(%)**				< 0.001
≥Once/month	2,029 (20.8)	1,751 (21.4)	276 (17.8)	
< Once/month	819 (8.4)	707 (8.6)	111 (7.1)	
Never	6,898 (70.8)	5,729 (70.0)	1,167 (75.1)	
Sleep time, hours, mean (SD)	6.4 (1.9)	6.4 (1.9)	6.2 (1.9)	0.001
**Physical_activity (%)**				
Intensive physical	367 (13.8)	313 (14.0)	54 (13.2)	0.496
Light physical	1610 (60.7)	1351 (60.3)	259 (63.3)	
Moderate physical	674 (25.4)	578 (25.8)	96 (23.5)	
**Clinical Measurements**				
SBP, mmHg, mean (SD)	129.4 (21.5)	128.6 (21.4)	133.6 (21.3)	< 0.001
DBP, mmHg, mean (SD)	75.2 (12.2)	74.8 (12.2)	77.0 (11.7)	< 0.001
Glucose, mg/dL, mean (SD)	109.6 (35.9)	103.1 (17.5)	144.8 (71.3)	< 0.001
BMI, kg/m^2^, mean (SD)	23.9 (28.6)	23.7 (31.1)	24.8 (4.0)	0.193
WC, cm, mean (SD)	84.0 (12.3)	83.2 (11.9)	88.5 (13.1)	< 0.001
TG, mg/dL, mean (SD)	131.9 (105.7)	124.9 (93.0)	169.6 (151.8)	< 0.001
HDL-C, mg/dL, mean (SD)	51.4 (15.5)	52.2 (15.3)	47.0 (15.5)	< 0.001
**CumAIP**				
CumAIP, mean (SD)	1.1 (0.8)	1.0 (0.8)	1.5 (0.9)	< 0.001
**CumAIP quartiles, n (%)**				< 0.001
Q1	1,745 (26.0)	1,590 (28.4)	155 (14.0)	
Q2	1,698 (25.3)	1,466 (26.2)	232 (21.0)	
Q3	1,679 (25.1)	1,388 (24.8)	291 (26.3)	
Q4	1,577 (23.5)	1,149 (20.5)	427 (38.6)	

NDM, Newly Diagnosed Diabetes; SBP, Systolic Blood Pressure; DBP, Diastolic Blood Pressure; BMI, Body Mass Index; WC, Waist Circumference; TG, Triglycerides; HDL-C, High-Density Lipoprotein Cholesterol; CumAIP, Cumulative Atherogenic Index of Plasma.

Physical activity data is omitted due to high proportion of missing values (only 2,651 participants had data). P-values for continuous variables were derived from t-test or ANOVA, and for categorical variables from χ^2^ test. Percentages for drinking and smoking status are calculated based on non-missing values.

### 3.2 Dose–response relationship between CumAIP and new-onset diabetes

Table 2 demonstrates graded increases in diabetes risk across ascending CumAIP quartiles (*p* for trend < 0.001). After full adjustment for sociodemographics (age, gender, education, location, marital status), lifestyle factors (smoking, drinking, sleep duration, physical activity time), and blood pressure (SBP/DBP), participants in the highest quartile (Q4) had a 3.43-fold elevated diabetes risk vs. Q1 (OR = 3.82, 95% CI: 3.13–4.67). Per interquartile range (IQR) increase in CumAIP as a continuous variable conferred a 90% higher diabetes risk (OR = 1.90, 95% CI: 1.75–2.07). CumAIP showed moderate predictive accuracy (AUC = 0.64, 95% CI: 95% CI for AUC: 0.63,0.66) with an optimal cutoff of 1.03 for risk stratification.

**Table 2:** Association of CumAIP with the risk of new-onset diabetes in the CHARLS.

**Parameter**	**Model 1**	***p***	**Model 2**	***p***	**Model 3**	***p***
CumAIP per IQR	1.90 [1.75, 2.07]	< 0.001	1.83 [1.56,2.15]	< 0.001	1.79 [1.52, 2.12]	< 0.001
**Quartiles of CumAIP**						
Q1	Ref		Ref		Ref	
Q2	1.62 [1.31,2.01]	< 0.01	1.58 [1.08, 2.33]	< 0.001	1.53 [1.02, 2.09]	< 0.01
Q3	2.15 [1.75, 2.65]	< 0.01	1.81 [1.25, 2.65]	< 0.01	1.75 [1.19, 2.61]	< 0.01
Q4	3.82 [3.13, 4.67]	< 0.001	3.64 [2.54, 5.27]	< 0.001	3.52 [2.42, 5.19]	< 0.001
*p* for trend	< 0.001		< 0.001		< 0.001	

Model 1 was crude model. Model 2 was adjusted for age, gender, education level, location, and marital status. Model 3 was adjusted for age, gender, education level, location and marital status, smoking status, drinking status, sleep time, Physical activity time, SBP, and DBP. CumAIP, cumulative atherogenic index of plasma; IQR, interquartile range; CHARLS, China Health and Retirement Longitudinal Study; SBP, systolic blood pressure; DBP, diastolic blood pressure.

**Figure 3:**
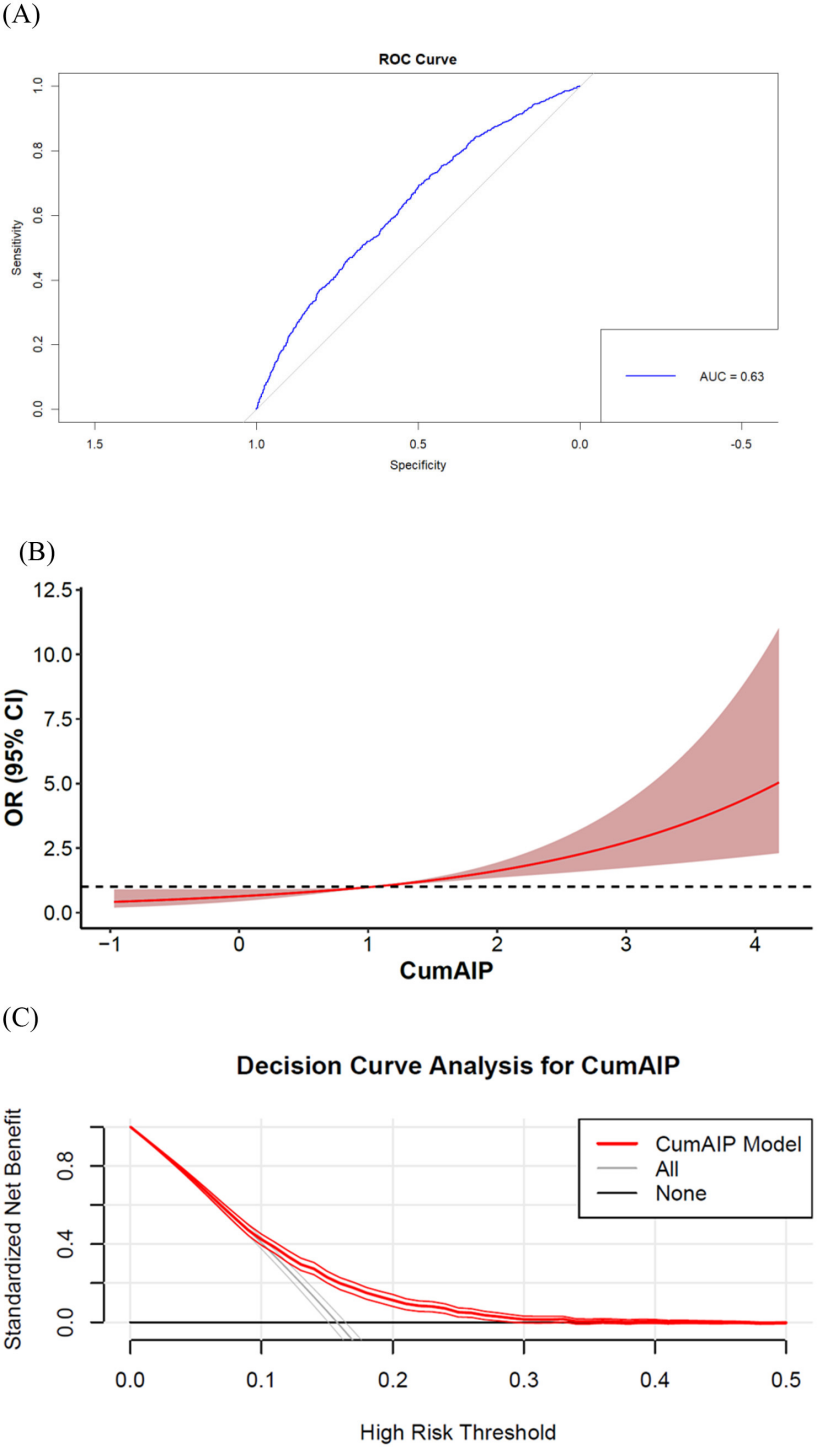
Predictive performance of cumulative atherogenic index of plasma (CumAIP) for new-onset diabetes. **(A)** ROC curve (AUC = 0.64, 95% CI: 0.63–0.66). **(B)** Dose-response relationship by restricted cubic splines (4 knots). **(C)** Clinical utility assessment by decision curve analysis. CumAIP demonstrates moderate discriminative ability with a linear risk gradient.

To evaluate the predictive value and dose-response relationship of CumAIP for new-onset diabetes, we analyzed model performance as shown in Figure 3A. Receiver operating characteristic (ROC) analysis demonstrated that CumAIP had moderate discriminative ability, with an area under the curve (AUC) of 0.64 (95% CI: 0.63–0.66). At the optimal cutoff value, the model achieved a sensitivity of 68.1%, specificity of 53.4%, positive predictive value (PPV) of 21.4%, and negative predictive value (NPV) of 89.9%. Decision curve analysis (DCA) further indicated the clinical utility of CumAIP across a range of threshold probabilities. The optimal cutoff value for risk stratification was determined to be 1.03 based on the maximum Youden Index. Dose-response relationship (Panel B) was examined using RCS regression, which confirmed a linear association between CumAIP and new-onset diabetes risk (p for non-linearity = 0.44). Notably, the optimal cutoff (1.03) from ROC analysis corresponded to a threshold where the cumulative diabetes risk began to increase gradually (as shown in Panel B), further supporting the clinical relevance of this cutoff. Subsequent piecewise linear regression (Supplementary Table 1) focused on CumAIP ≥1.03 and found that each IQR increase was associated with a 90% higher risk (OR=1.90, 95% CI: 1.74–2.06).

The optimal CumAIP cut-off value of 1.03 corresponded to specific lipid profiles that can guide clinical practice. Based on the inverse transformation of AIP [AIP = log10(TG/HDL-C)], this threshold translates to a TG/HDL-C ratio of approximately 10.47 (Table 3).

**Table 3:** Assuming median values from our cohort.

**Parameter**	**mg/dL units**	**mmol/L units**	**Conversion factor**
TG/HDL-C ratio	10.47	10.47	1.00
Corresponding TG^*^	157.1	1.77	TG:1 mmol/L = 88.57 mg/dL
Corresponding HDL-C^*^	15.0	0.39	HDL-C:1 mmol/L = 38.67 mg/dL

TG, triglycerides; HDL-C, high-density lipoprotein cholesterol.

**Figure 4:**
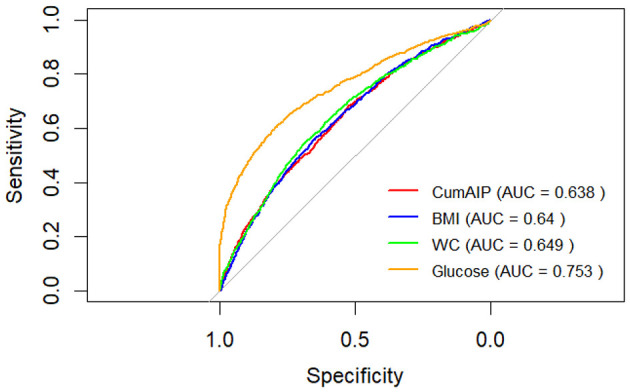
AUC curves of different prediction models.

To demonstrate the differences between CumAIP and traditional diabetes predictive indicators such as BMI, WC, and fasting blood glucose. First, we calculate the AUC values of BMI, WC, and fasting blood glucose respectively. The AUC value of BMI is 0.64. The AUC value of WC is 0.65. The AUC value of fasting blood glucose is 0.75 (Figure 4). Secondly, we conducted research to construct three prediction models and evaluated their prediction performance through AUC. The results indicated that the AUC of the traditional factor model (BMI+WC + fasting blood glucose + age + gender) was 0.7729 (95% CI: 0.75–0.79), suggesting that this model has a good predictive ability for outcome events. The AUC of the CumAIP standalone model was 0.64 (95% CI: 0.62–0.66), close to the AUC=0.63 mentioned by the reviewers, and its predictive efficacy was significantly lower than that of the traditional factor model.

The AUC of the combined model (traditional factors + CumAIP) was 0.78 (95% CI: 0.76–0.79), slightly higher than that of the traditional factor model, but the improvement was very small and there was no obvious advantage. The classification NRI was −0.0003 (95% CI: −0.0144 to 0.0138), *p* = 0.97012; The continuous NRI was 0.05 (95% CI: −0.02 to 0.11), *p* = 0.16; The IDI was 0.0009 (95% CI: −0.0005 to 0.0024), *p* = 0.20125 (Table 4).

**Table 4:** Comparison table of AUC values and 95% confidence intervals of different prediction models.

**Model**	**Predictors**	**AUC**	**(95% CI)**
Traditional factors only	BMI + WC + Glucose + Age + Sex	0.77	0.75–0.79
CumAIP only	CumAIP	0.63	0.62–0.66
Combined model	All variables	0.77	0.76–0.79

BMI, Body Mass Index; WC, Waist Circumference; CumAIP, Cumulative Atherogenic Index of Plasma.

### 3.3 Hierarchical analysis

**Figure 5:**
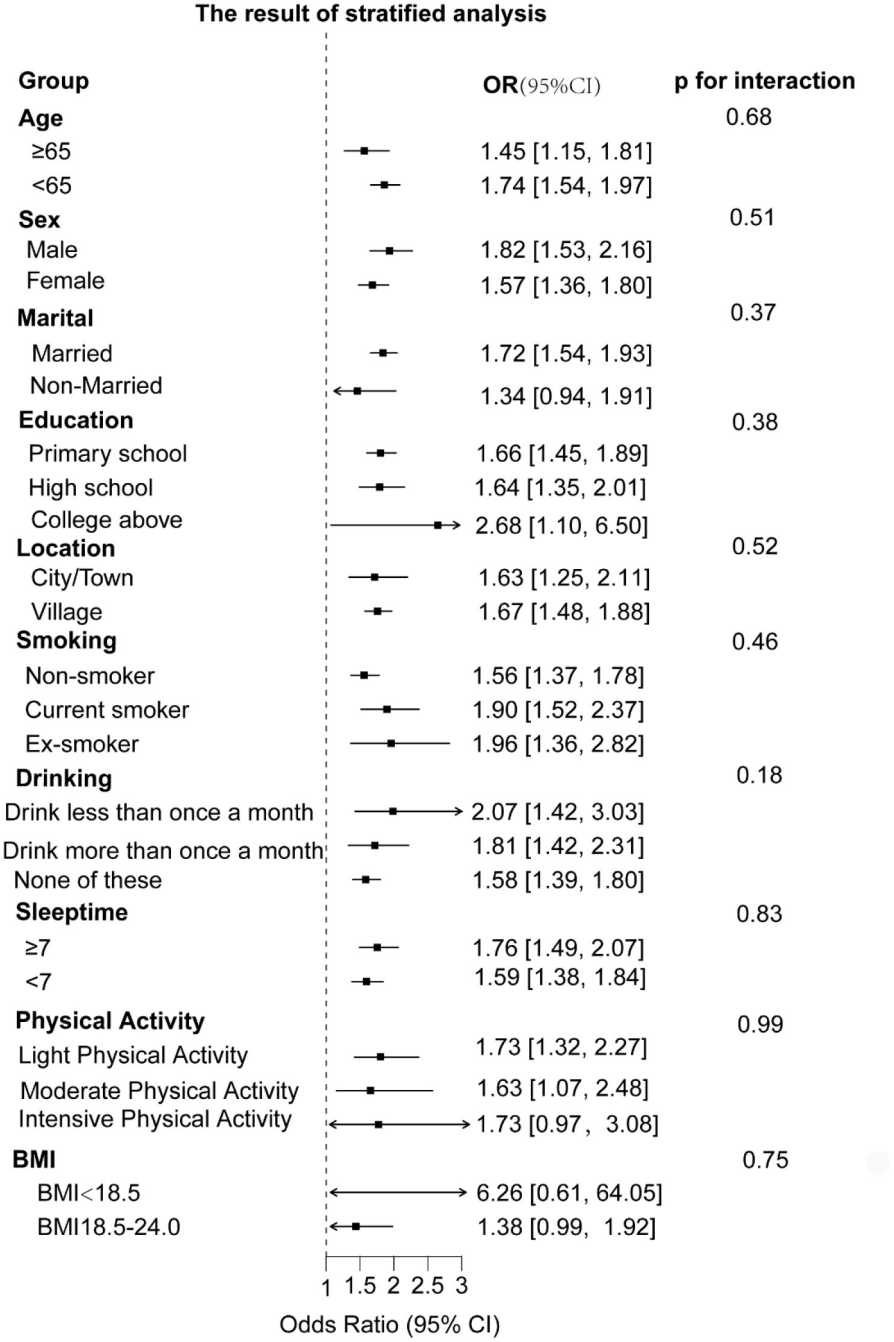
The result of stratified analysis.

To understand whether CumAIP would have different effects on the risk of new-onset diabetes in different subgroups, we grouped the participants by characteristics. The results showed that the impact of CumAIP on the risk of new-onset diabetes was consistent among the subgroups. The *P*-values of all interactions were >0.05 (ranging from 0.16 to 0.99), indicating that among all stratified factors, there was no statistically significant difference in the association between exposure and outcome among different subgroups (Figure 5).

### 3.4 Methodological considerations in cumulative exposure assessment

**Figure 6:**
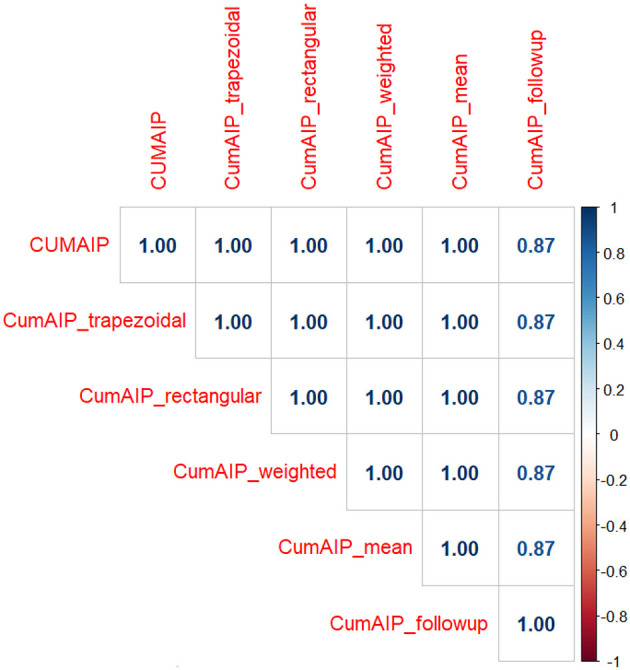
Correlation Matrix of Alternative Cumulative AIP Formulations. Heatmap displaying Pearson correlation coefficients between different methods of calculating cumulative atherogenic index of plasma (CumAIP). The perfect correlations (r = 1.00) among the first five methods confirm mathematical equivalence in our two-timepoint study design. The follow-up-only approach shows high but imperfect correlation (r = 0.87).

Our sensitivity analysis addresses an important methodological consideration raised by prior research regarding cumulative exposure quantification. The perfect concordance among alternative formulations (Figure 6) stems from mathematical equivalence in two-timepoint designs, where all methods reduce to linear combinations of the baseline and follow-up measurements. This finding has two key implications: First, it confirms the robustness of our results to methodological variations in exposure quantification. The consistent effect estimates across formulations suggest that our conclusions regarding the CumAIP-diabetes association are not artifacts of specific mathematical choices. Second, it highlights the context-dependent nature of cumulative exposure metrics. While methodological distinctions would be more consequential in studies with more frequent measurements, our findings provide reassurance for similar two-timepoint investigations in nutritional epidemiology. The lower correlation observed with the follow-up-only approach (r = 0.875) underscores the value of incorporating longitudinal measurements, as baseline AIP contributes meaningfully to cumulative exposure assessment beyond follow-up values alone.

## 4 Discussion

In this prospective cohort study involving middle-aged and elderly people in China, we determined for the first time that CumAIP exposure is an important risk factor for the occurrence of diabetes and increases the risk of diabetes. Furthermore, compared with married individuals, being unmarried can reduce the risk of CumAIP diabetes.

Based on the baseline data of CHALS, we found that more newly diagnosed diabetic patients were female. The newly diagnosed population lives in rural areas there were more patients who had never smoked SBP, DBP, WC, glucose, TG and CUMAIP were higher than baseline levels, and HDL-C was lower Based on the follow-up data we found that CUMAIP was positively correlated with the risk of NDM. After adjusting for confounding factors, for each increase in IQR of CUMAIP, the risk of NDM increased by 90% (OR=1.90, 95% CI: 1.74–2.06). Based on the dose-response relationship analysis we found that there was a linear relationship between CumAIP and the risk of NDM. Previous follow-up studies have shown that there is a nonlinear relationship between AIP and prediabetes and diabetes (15). However, our results show a linear correlation between cumAIP and new-onset diabetes. This may be related to the different pathological mechanisms at different stages of the disease. In prediabetes, β cells can still maintain blood glucose homeostasis through compensation. At this time, CumAIP may only trigger metabolic decompensation above a specific threshold, thus showing nonlinearity. However, new-onset diabetes is marked by β -cell failure (16). At this time, the atherosclerotic effect of CumAIP may run through the entire risk spectrum, so the result is linear (17).

This study systematically evaluated the incremental value of the cumulative atherosclerosis index (CumAIP) in the risk prediction of diabetes. Although the integrated model demonstrated slightly higher discriminative ability than traditional factors, neither the net reclassification improvement (NRI) nor the comprehensive discriminative improvement (IDI) reached statistical significance. This discovery may have several explanations: firstly, traditional risk factors (such as BMI and fasting blood glucose) are already strong predictors in themselves, leaving limited room for incremental improvement in new indicators. Secondly, the atherosclerotic lipid metabolism pathway represented by CumAIP may share some pathophysiological mechanisms with traditional factors, resulting in limited independent information it provides. However, this does not mean that CumAIP has no clinical value. Its unique advantage lies in capturing the long-term cumulative burden of lipid metabolism, which may be of great significance for identifying high-risk individuals whose traditional risk factors are at a critical level but have long-term abnormal lipid metabolism. Future research can explore the predictive value of CumAIP in specific subgroups, such as non-obese people.

According to the results of Hierarchical Analysis, Stratified analyses demonstrated consistent positive associations between CumAIP and diabetes risk across all predefined subgroups (Figure 3). Notably, the association remained statistically significant in most strata, including different age groups, genders, education levels, and lifestyle factors. Some subgroups (such as those non-married, those with a college degree or above, those with intensive physical activity, and those with a low BMI) may have lower statistical power due to insufficient sample size formal tests for interaction revealed no significant effect modification by any of the examined characteristics (all P-interaction > 0.05) suggesting the robustness of the CumAIP-diabetes association across diverse demographic and behavioral profiles.

Our study has several limitations that should be acknowledged. First of all, the ADA criteria are the most inclusive, while the International Expert Committee and WHO criteria are more restrictive (18). Although the diagnostic criteria for diabetes refer to the ADA standards, they mainly rely on self-reports and fasting blood glucose without conducting OGTT, which may underestimate the incidence of diabetes. In future studies, it is considered to incorporate multiple indicators such as HbA1c and OGTT for a clear diagnosis. Secondly, the participants of the CHARLS cohort and our analytic sample were predominantly of Han Chinese ethnicity. In previous studies, only Chinese people recorded data on this content, without involving ethnic differences, which has certain racial limitations.

Lack of inflammatory markers (e.g. CRP) precluded exploration of inflammation as a potential mediator. Diabetes classification relied on biomarkers and self-report without distinguishing type, though the cohort age suggests most were type 2 (19). Residual reverse causality from subclinical diabetes influencing lipid levels cannot be entirely ruled out. Despite this, the study's prospective design, large representative sample, and novel investigation of cumulative AIP provide a valuable foundation for future work.

The perfect inter-method correlations observed in our sensitivity analysis (Figure 3) provide strong evidence for the robustness of our cumulative exposure metric. While methodological choices may exert greater influence in studies with more frequent measurements, our two-timepoint design ensures result invariance to the specific mathematical formulation employed.

Although the diagnostic criteria for diabetes refer to the ADA standards, they primarily rely on self-reporting and fasting blood glucose without performing an OGTT, which may underestimate the incidence rate of diabetes. This limitation should be pointed out in the discussion, and it is recommended that future studies incorporate multiple indicators such as HbA1c and OGTT for definitive diagnosis.

Our findings on the positive association between cumulative CumAIP exposure and diabetes incidence reinforce and extend the growing body of evidence linking atherogenic dyslipidemia to glucose metabolic disorders. A research demonstrated that high CumAIP exposure increases the risk of progression from prediabetes to diabetes (10). However, our study provides several critical advancements. First, we investigated this relationship in a broader, community-based cohort of adults free of diabetes at baseline, thereby highlighting the role of CumAIP in the primary prevention of diabetes, not solely in the progression of pre-existing prediabetes. Second, with a longer follow-up period of seven years, our assessment of the cumulative effect of atherogenic burden is more robust. Most importantly, and to the best of our knowledge, this is the first study to formally evaluate the predictive performance of CumAIP for new-onset diabetes. We identified a specific cut-off value and demonstrated a moderate predictive ability (AUC = 0.64), presenting a tangible metric for future risk stratification strategies. Furthermore, our dose-response analysis precisely quantifies that each unit increase in CumAIP is associated with a 90% elevated risk, providing a clear message for clinical communication. Therefore, while confirming the association established in narrower populations, our work significantly advances the field by underscoring the clinical utility of CumAIP as a potential screening and risk assessment tool”.

CumAIP has significant potential in clinical application and prognosis evaluation. China is currently one of the countries with the heaviest burden of atherosclerotic diseases in the world. Therefore, it is particularly important to use simple detection indicators to quantify the accumulated atherosclerotic exposure (20, 21). We believe that incorporating CumAIP into clinical practice may help reduce the burden of atherosclerotic diseases and provide information for the prevention of diabetes. It is suggested that government agencies establish health consultation centers for middle-aged and elderly people in communities and rural areas, strengthen health education and enhance the awareness of the importance of CumAIP.

## 5 Conclusion

This prospective cohort study targeting middle-aged and elderly people in China indicates that CumAIP is closely related to new-onset diabetes. High CumAIP exposure increases the risk of diabetes. These findings suggest that monitoring and maintaining appropriate AIP levels may help prevent the deterioration of blood sugar levels.

## Data Availability

The datasets presented in this study can be found in online repositories. The names of the repository/repositories and accession number(s) can be found in the article/Supplementary material.
