# Supplementary material for: Cumulative atherosclerosis index of plasma exposure and new-onset diabetes in middle-aged and older adults: a prospective cohort analysis from the China Health and Retirement Longitudinal Study
Source: Front Nutr. 2025 Oct 29;12:1653764. doi: 10.3389/fnut.2025.1653764 (PMC12605295; doi:10.3389/fnut.2025.1653764)
Supplement: Supplementary file 1 [file Data_Sheet_1.docx]

1.Supplementary Material S1: Mathematical Derivation of Cumulative Exposure Equivalence

In a two-timepoint study design with measurements at t₁ and t₂, the cumulative exposure is conceptually defined as the definite integral of the concentration function over time.

Cumulative Exposure = ∫[t₁ to t₂] C(t) dt

For discrete measurements, this integral can be approximated using numerical integration methods:

1. Trapezoidal Rule (Primary Method):

[(C(t₁) + C(t₂)) × (t₂ - t₁)] / 2

2. Rectangular Rule (Average Value Method):

Mean(C) × (t₂ - t₁) = [(C(t₁) + C(t₂)) / 2] × (t₂ - t₁)

3. Time-Weighted Average:

[C(t₁) × t₁ + C(t₂) × t₂] / (t₁ + t₂) × (t₂ - t₁)

In the specific case of two timepoints with a constant time interval, these three formulations reduce to mathematically equivalent linear combinations of C(t₁) and C(t₂), differing only by a constant scaling factor.

2.Supplementary Table 1.Associations of CumAIP with the risk of new-onset diabetes using Piecewise linear regression.

|  | **Model 1** | ***P*** | **Model 2** | ***p*** | **Model 3** | ***p*** | |
| --- | --- | --- | --- | --- | --- | --- | --- |
| CumAIP per IQR |  |  |  |  |  |  |  |
| ≥1.03 | 1.90 [1.74, 2.06] | <0.001 | 1.90[1.74,2.06] | <0.001 | 1.70 [1.42, 2.03] | <0.001 |  |

Model 1 was crude model. Model 2 was adjusted for age, gender, education level, location, and marital status. Model 3 was adjusted for age, gender, education level, location and marital status, smoking status, drinking status, sleep time, physical activity time，SBP, and DBP.

CumAIP, cumulative atherogenic index of plasma ; IQR, interquartile range; SBP, systolic blood pressure; DBP, diastolic blood pressure.

3.Supplementary Material S2:**Data availability**

The datasets supporting the conclusions of this article are available on the CHARLS website: <http://charls.pku.edu.cn/>.
